# A Global Systematic Review and Meta‐Analysis of Methods Used to Evaluate Predation and Diet of Domestic Cats (*Felis catus*)

**DOI:** 10.1002/ece3.71349

**Published:** 2025-04-25

**Authors:** Hannah L. Lockwood, Maren Huck

**Affiliations:** ^1^ College of Science and Engineering University of Derby Derby UK

**Keywords:** cats, depredation, detectability, diet, meta‐analysis, palatability, predation

## Abstract

Invasive species, including multiple domestic species, can devastate local biodiversity. Domestic cats ( *Felis catus* ) can cause declines in select prey species around the world, and multiple methods are employed to monitor cat diet and predatory habits. These methods have not yet been compared against one another in a meta‐analytical way, and therefore, the aim here was to evaluate the relative proportions of different taxa reported in the cat diet. We compared 88 studies using a beta regression model conducted on four different taxa, where methodology, location and duration of study were included as variables. Mammals were further divided into rodents, insectivores and medium‐sized mammals for a subset of European studies, using Wilcoxon Rank Sum tests to compare methods. Proportions of mammals were lowest, and those of herptiles were highest in studies using collar‐mounted cameras. However, greater proportions of birds were recorded in return questionnaires, suggesting detectability bias, as bird remains are easier to detect. Mammal figures were lower in Australasian studies, whereas birds were more frequently reported in Australasia than in other mainland locations, likely reflecting a difference in prey availability. In Europe, insectivores were found to be more frequently returned than eaten, supporting the existing hypothesis that this group is largely unpalatable to cats. Care should be taken when extrapolating data gathered by different methods, as each one fundamentally measures a different aspect of diet. Only six (6.8%) studies here used video cameras and, although a useful monitoring technique, video results showed a different pattern in taxonomic proportions to data gathered using consumed or returned prey. More research using cat cameras in locations of varying faunal composition is necessary, improving the general applicability of video data to cat populations globally. Palatability and detectability of prey appear to influence the data reported, and these aspects should be considered when calculating total predation rates.

## Graphical abstract

Domestic cats ( *Felis catus* ) have the potential to negatively affect prey populations, but in order to assess impact, accurate dietary data must first be gathered. The methods used to estimate predation by cats can affect the results produced, with cat‐cam video studies reporting higher proportions of reptiles and lower mammal figures than other, more traditional methods. More camera research is needed globally to improve the reliability and applicability of estimates produced.

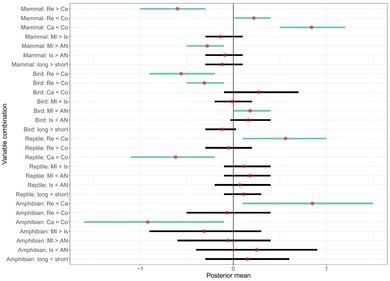

## 1 Introduction

Invasive species are one of the largest threats to biodiversity and can affect local systems in a number of ways, including disease transmission, competition for resources, hybridisation and direct depredation (JNCC 2024). Invasive species are defined here as species which occur outside of their natural range (commonly facilitated by human movement) and have deleterious effects on local, native ecosystems (Doherty et al. 2016). Free‐roaming domestic animals, such as dogs ( *Canis familiaris* ) and goats ( *Capra hircus* ), are commonly amongst the most invasive species, having a large negative impact on local flora and fauna (Medina et al. 2011). Domestic cats ( *Felis catus* ) are now cited as contributing to the decline and extinction of 367 and 63 species, respectively, according to a meta‐analysis comparing the impacts of invasive mammalian predators (Doherty et al. 2016). Their effects as predators are particularly apparent on oceanic islands, where high species endemism and poorly developed predator defence strategies can lead to severe declines and extinctions of native species (Medina et al. 2011; Lepczyk et al. 2023). Endemic species are particularly vulnerable, as population size may not be robust enough to sustain predation by cats. In a review by Lepczyk et al. (2023), it was found that nine endemic island species, known to be domestic cat prey, are now listed by the IUCN as Extinct or Extinct in the Wild. However, in Europe, prey species have evolved alongside the closely related European wildcat ( *F. silvestris* ) and mustelid species, exerting similar pressure on populations (Beaumont et al. 2001; Clegg 2017). Therefore, prey present in these areas are likely better adapted to either avoid predation (through crypsis, escape, or defence) or able to cope with losses (through high productivity rates) than on remote islands where prey species have not faced such predation pressures previously (Bradshaw et al. 2012; Clegg 2017). Indeed, Lepczyk et al. (2023) found that 74.8% of species consumed on islands were not of particular conservation concern, whereas this percentage increased to 91.4% on continental mainlands. In Europe, for example, 86.3% of consumed prey (or scavenged individuals) were found to be listed as Least Concern by the IUCN (Lepczyk et al. 2023), and in a recent study in the UK, only 0.21% of returned prey were classed as European Protected Species (Lockwood et al. 2025).

Of course, not all animals appearing in the diet of cats have been caught by them. For example, large‐bodied species (such as deer) and medium‐sized carnivores (including other cats) are occasionally scavenged and reported in studies of consumed individuals (Jones 1977; Biró et al. 2005). Although scavenging is thought to be rare, since most of the cats' protein and water intake comes from fresh kills (Read et al. 2018), high proportions of the cats' diet were thought to be scavenged in a South African island study (88.6% of birds eaten were scavenged, Apps 1983). In addition, not all animals caught by cats are eaten and, therefore, cannot be described as part of the cats' diet. For ease of reading, here, dietary items and those returned by cats are referred to as ‘prey’, as is common practice in the field.

In order to monitor the potential impacts of predation by cats, the number of prey caught must somehow be estimated. Often, owned pet cats are monitored using owner surveys, recording prey returned home (e.g., Churcher and Lawton 1987; Robertson 1998; Woods et al. 2003; Kays and DeWan 2004; Baker et al. 2005; Thomas et al. 2012; McDonald et al. 2015). Such survey data can then be multiplied by some factor to give an estimate of total predation, since cats are unlikely to carry home all prey. For example, a multiplication factor of 3.3 is suggested by Kays and DeWan (2004), based on direct observation of cats returning 30% of all prey caught (during the summer). When extrapolating data to produce an estimate of total predation, previous studies generally group all prey together (Baker et al. 2005; Maclean et al. 2008; Thomas et al. 2012). For example, Maclean et al. (2008) based their extrapolation of bird predation on the 3.3 multiplier by Kays and DeWan (2004), despite this factor being developed using mostly mammal data. A multiplication factor which is disproportionately or wholly based on mammals (particularly rodents) could over‐ or under‐estimate predation of other taxa, since prey taxa are likely returned in different proportions (Loyd et al. 2013; Seymour et al. 2020; Lockwood 2024). Indeed, Loyd et al. (2013) found that mammals were returned 50% of the time, whereas reptiles were returned on only 7.14% of occasions, suggesting that if all prey data were extrapolated using the same multiplier, mammal predation would be slightly over‐estimated, and reptiles vastly under‐estimated.

The accuracy of multiplication factors is also affected by the detectability of different prey groups in studies using different methods. For example, the remains of some taxa (such as invertebrates) may be less detectable in gut and scat analyses, since the exoskeleton can be easily broken down throughout digestion (Woolley et al. 2020). Similarly, in studies of cat owners monitoring prey returned, the remains of larger‐bodied prey and birds may be more obvious and observable than those of small mammals, as for birds, feathers are commonly left at the site of capture or consumption (Lockwood et al. 2025). This detection bias may lead to more pronounced under‐reporting of small mammal prey than that of birds, for example. Therefore, returned bird values recorded are potentially closer to the true capture rates than those of small mammals, although this matter does require further investigation. In addition, some prey groups may be more palatable to cats than others, with insectivores being particularly unpalatable (Toner 1956; Turner and Bateson 2000; Krauze‐Gryz et al. 2012; Kauhala et al. 2015; Lockwood et al. 2025). Indeed, Krauze‐Gryz et al. (2012) found that insectivores represented 11.8% of the returned diet of cats, yet only 1.1% of eaten prey. It is therefore important to disentangle the effect of methodology on different types of prey species.

Cat dietary studies can be divided into three key groups: return surveys (owner questionnaires), consumption studies (scat and stomach analyses) and video capture studies (from collar‐mounted cameras). Return surveys have traditionally been an effective and popular method to monitor pet cat prey, since they are non‐invasive and only require consent from participants but do not require landowner permissions (since researchers do not need access to land) or extensive fieldwork (e.g., Churcher and Lawton 1987; Woods et al. 2003; Baker et al. 2005; Kauhala et al. 2015; Castañeda et al. 2020). There can, however, be problems with prey identification if researchers cannot confirm the accuracy of identification by cat owners. Return methods do not account for prey discarded or eaten by cats unless this is witnessed by owners and recorded.

Consumption studies use either scat or stomach and gut analysis to examine items eaten by cats (e.g., Nilsson 1940; Eberhard 1954; Brickner‐Braun et al. 2007; Krauze‐Gryz et al. 2012; Piontek et al. 2021). Whilst scat samples can potentially be collected for both feral and owned cats (depending on whether faeces are buried or exposed), stomach analysis is conducted post‐mortem and, therefore, is usually conducted on feral individuals (but, see Piontek et al. 2021). Of course, these consumption methods do not take into account prey animals that are killed and discarded or that escape, perhaps fatally injured.

Video cameras attached to a cat's collar can be employed to produce an accurate estimate of daily predation, although the battery life of these devices can be variable (Loyd et al. 2013; McGregor et al. 2015; Hernandez et al. 2018; Bruce et al. 2019). There is, indeed, a possibility that video cameras (or any other device attached to the cats themselves) could impact and limit the cats' ability to exhibit natural behaviours, including hunting effectively. However, Coughlin and van Heezik (2015) suggest that devices should not exceed 2% of a cat's body weight, so even the larger cameras used in such studies (e.g., 90 g, Loyd et al. 2013) may be suitable for cats weighing around 4.5 kg, although device dimensions should be considered also. Camera traps (or trail cameras) have also been used to monitor predation by cats, but this method does not give an idea of overall cats' diet, as it is limited by each camera's field of view (Herrera et al. 2022).

Whilst there are advantages and disadvantages of each, these dietary methods fundamentally measure different things: prey carried home (return surveys), prey eaten (consumption studies) and total prey caught (video capture studies). Although not often compared, where research has measured more than one of these aspects of cat diet, differences between taxonomic groups have been apparent. For example, a study in Poland found that shrews were more frequently returned than eaten, yet the reverse pattern was seen in invertebrates (Krauze‐Gryz et al. 2012). Similarly, another study, also conducted in Poland, showed invertebrates to be far more prevalent in consumption records (making up 27.2% of all prey individuals) than in return records (0.3% of all prey, Piontek et al. 2021). These examples suggest that some taxa are more frequently returned than eaten, and vice versa, which may cause under‐ and over‐representation of some taxa in extrapolated data. Since these potential differences in how each taxon is treated by cats (i.e., eaten, discarded) have not yet been examined in a meta‐analytical way, further examination of taxonomic differences is warranted.

Further to these taxonomic considerations, as most consumption studies (although not all) are conducted on feral individuals, dietary data generated using these methods cannot reasonably be applied to pet cats, as this would cause an inflated estimate of predation and impact on wild populations. Similarly, return studies cannot be conducted on feral cats and so all concern owned pet cats. Due to this relationship between methods used and cat type (feral or owned), the results of each approach should not be used interchangeably.

The location of a study may also influence the prey proportions reported. Since cats are generalist predators, hunting in accordance with prey availability (Churcher and Lawton 1987; Molsher et al. 1999; Loyd et al. 2013; Kitts‐Morgan et al. 2015; Yip et al. 2015; Krauze‐Gryz et al. 2017; Read et al. 2018; Lepczyk et al. 2023), taxa reported in dietary studies will likely depend on local prey availability and relative prey population sizes. For example, despite being home to a number of introduced species, New Zealand does not have any native terrestrial mammal species (only bats, IUCN 2020). Therefore, it can be expected that research in Australasia may report lower mammal proportions than elsewhere, owing to a difference in faunal composition. Similarly, habitat and climate will affect the prey distributions, such that reptiles, for example, may be more prevalent in warmer, drier climates. Whilst some studies use data from a range of locations to extrapolate and form conclusions (e.g., Loss et al. 2013 used data from multiple temperate regions to estimate US predation rates), this is not always appropriate, as predation rates and prey proportions likely vary depending on local fauna availability. Therefore, it is important to examine any differences in results from different geographical areas.

Prey availability also varies by season (Merritt 2010; Holden and Cleeves 2014), such that studies monitoring diet over a short period are unsuitable for extrapolation across a full year (see Marra et al. 2015). As the breeding season for most birds and mammals begins in the spring (Hume et al. 2016; Couzens et al. 2017), predation by pet cats (although not necessarily feral cats) will likely be higher during the spring and summer periods than the winter months (Lockwood et al. 2025). Similarly, many herptile species, particularly in cooler climates, hibernate over the winter period and are therefore less available to cats during this time (Speybroeck et al. 2016). Invertebrate activity also increases with the warmer climatic conditions of the spring and summer, as many remain in their larval form over the winter (Martay and Pearce‐Higgins 2018; Shaftel et al. 2021). It should, however, be noted that seasonality is linked closely with location, with some populations increasing and others decreasing in the winter as species migrate to and from different parts of the world. For example, whilst some bird populations are found in the UK only in the warmer months, such as reed warblers, *Acrocephalus scirpaceus* , others are found there only during the winter, such as redwings ( *Turdus iliacus* ; Holden and Cleeves 2014).

As not all prey species are of equal conservation concern in certain locations (e.g., invasive rats, *Rattus rattus* and *R. norvegicus,* and endemic reptiles), it is important to understand which factors influence the relative proportions of different taxa in the diet of domestic cats. Here, the primary aim was to examine the differences in taxa reported using three key methods (return surveys, consumption studies and video capture methods) in different locations around the world. Since the methods used in cat dietary research have not yet been comprehensively analysed (although briefly looked at by Lepczyk et al. 2023), data generated by each method should be compared. The second aim was to establish whether some prey groups may be more or less palatable than others, since there is some evidence to suggest that shrews are unpalatable to cats and therefore less likely to be eaten (Krauze‐Gryz et al. 2012; Lockwood et al. 2025).

We hypothesised that (i) for each taxonomic group (class), the proportions recorded would vary depending on the method used to collect the data. Specifically, we predicted that mammals would be more strongly represented in consumption studies, whilst birds and herpetofauna would be relatively more prevalent in return studies. In addition, (ii) since Australia and New Zealand do not share the faunal composition of areas such as mainland Europe and the United States (Inns 2009; Cogger 2018), we hypothesised that reported taxon proportions would also vary by location around the World, with higher proportions of birds predicted for Australasia and islands compared to mainland studies. We also hypothesised that (iii) when looking solely at mainland European studies, some prey groups would be eaten and returned home in different proportions, as some prey groups are thought to be particularly unpalatable to cats, with a predicted higher representation of insectivores and birds in return studies than consumption studies, and the opposite effect for rodents (Krauze‐Gryz et al. 2012).

## 2 Methods

**FIGURE 1:**
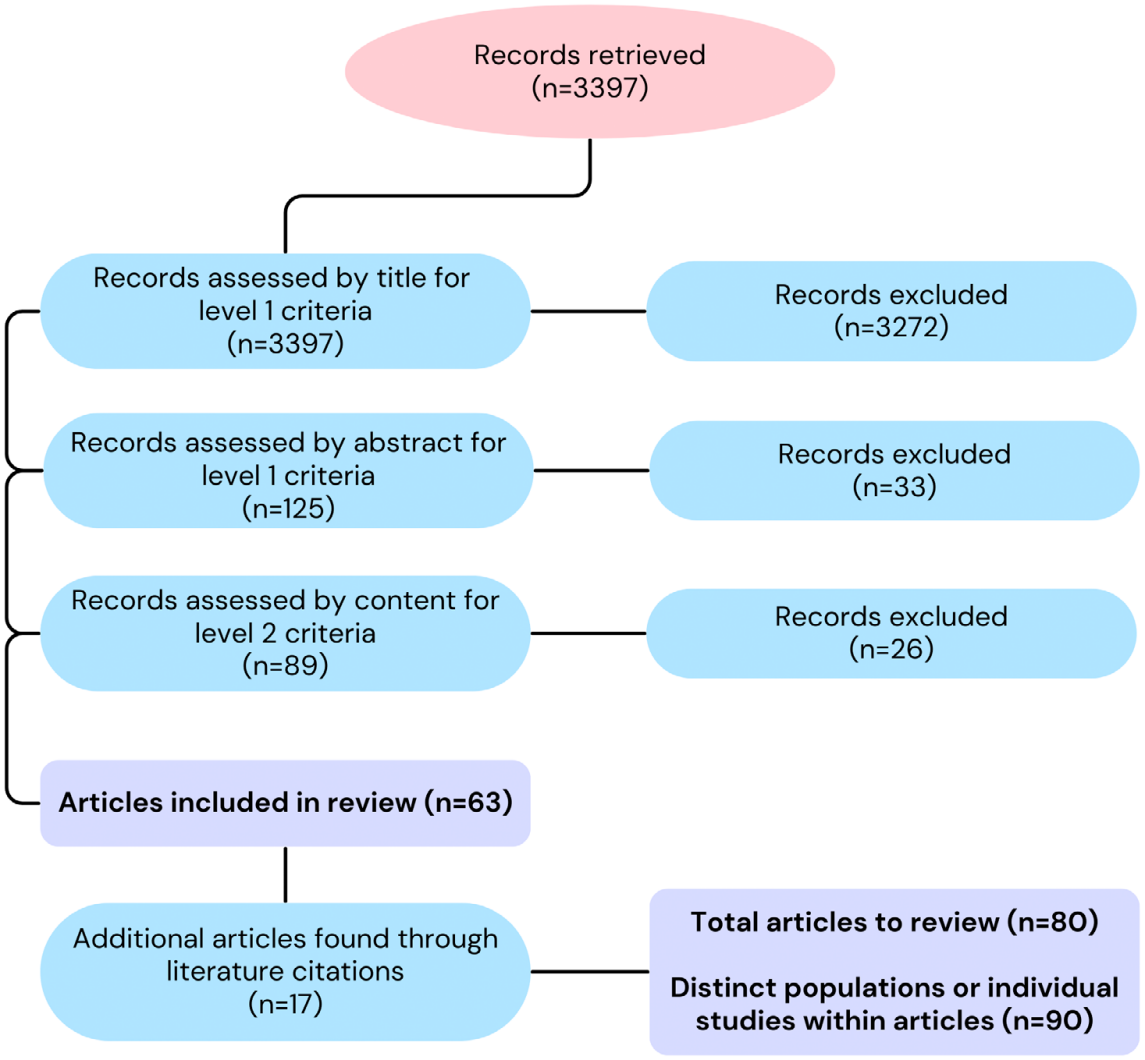
The process of study selection showing the number of articles retained and excluded at each stage.

In order to determine possible effects of various survey methods and location on estimates of the relative proportions of different prey taxa, we conducted a meta‐analytical study, combining findings from all relevant published scientific sources and following the PRISMA guidelines (Page et al. 2021). In February 2025, we used Web of Science (core collection) to search using the following terms: ALL = ((cat OR cats OR catus) AND (predat* OR prey OR threat OR kill OR risk* OR depred*) AND (wildlife OR bird* OR mammal* OR amphibian* OR reptile*)). There was no lower date limit applied when searching the database, so all dates (up to February 2025) were considered for inclusion. We then filtered the studies retrieved through this initial search by title and abstract, retaining those on prey preference, diet, predation rate, return rate and impact of cats on wildlife (considered ‘level 1 criteria screening’, Figure 1). Other topics retrieved by the search included big cat studies, canid studies and epidemiology and parasite studies, all of which were excluded here at this initial stage.

One researcher (independently to avoid any inclusion bias) then reviewed the remaining articles for a second time, this time searching the main body of each study (level 2 criteria, Figure 1). Level 2 screening required that studies gave a total number of prey captured (using the video approach), returned (questionnaires), or eaten (scat and stomach analyses), along with a breakdown of prey, either by taxon or, where available, species. Where consumption studies (scat and stomach) gave Relative Frequency of Occurrence (RFO, the number of occurrences of a prey type divided by total number of occurrences of all prey types × 100), data were recalculated to provide the number of prey consumed (*N* data). Frequency of Occurrence (FO, percentage of a scats or stomachs containing a prey type) and biomass (biomass ratio of each prey type) studies were excluded. Although FO and biomass are useful measures of consumption (Reynolds and Aebischer 1991) and are widely used (Nogales et al. 1988; Tidemann et al. 1994; Medina et al. 2008; Shionosaki et al. 2015), they are not comparable with prey caught or prey returned studies, since they do not express the total number of prey consumed. As the total number of prey returned, eaten and caught (as seen on camera footage) was needed here in order to investigate taxonomic differences and potential issues with previous extrapolation methods, it was important that only those studies reporting data in the described format were included. Therefore, biomass research was not useful in this instance, despite its clear relevance in other contexts. Other methods utilised in dietary research, such as the use of motion‐activated trail cameras (used by Herrera et al. 2022, for example), were also not included here. Trail cameras (or camera traps) produce data which are largely dependent on the placement of camera units and cannot give an overview of the total range of prey or number of individuals caught or consumed by each cat. Therefore, results can be biased towards certain prey groups, depending on camera location. Trail cameras are generally used to monitor which species depredate certain bird colonies, for example (Greenwell et al. 2019; Blanchard et al. 2024; Sadik and Akash 2024). However, since cats are not the focal species, but the prey, such studies do not give data as an estimate of total prey individuals per cat and were, therefore, not included here. Similarly, studies using eDNA were excluded, since they offer presence or absence data, along with relative eDNA concentrations, rather than the number of prey individuals (Plimpton et al. 2021).

For each study which met the described criteria, the method used, along with the location (mainland or island) and country code, duration of study, cat type (feral or owned), sample sizes (of cats or scat, and prey), proportion of diet made up by each taxon and citation information were recorded. Since data for invertebrates do not appear to have been consistently recorded (particularly in return questionnaires), we have omitted invertebrate data from analyses here. Where a study reported invertebrates in their results, we recalculated the proportions to exclude this group.

We combined scat and stomach methods into one category (consumed), giving three key methods used (returned as identified by questionnaires, consumed and caught via video footage). Where ‘herpetofauna’ was stated in a study's results (and not divided into amphibians and reptiles), we removed these data points from the analyses (two studies). We split location into three categories (island, mainland and Australia and New Zealand ‘AN’) to assess the influence on reported prey proportions, as Australasian faunal composition is known to differ from the rest of the world, with New Zealand having no native land mammals (IUCN 2020). For the purposes used here, an island was defined as being an area of land no larger than 100,000 km^2^, such that small oceanic islands were included, yet land masses like mainland Britain were categorised separately (as mainlands). We classified studies according to their duration, i.e., whether they lasted for at least 12 months, or less (two categories). Due to the seasonal variability of prey availability, overall duration is of high importance when compiling data for annual extrapolation. It was also noted whether cats in each study were owned (being intentionally provided with food and shelter) or feral (may be occasionally fed by people, but reside outdoors; descriptions are available in Table S1). There was a strong correlation between the methods used and cat type (feral or owned) (Pearson *R* = 0.65, df = 86, *p* < 0.001) and therefore, only one of these two factors would be considered for model inclusion. After comparing the AIC values for tests including either variable, the method used (returned, caught, or consumed) was included and cat type removed.

We tested the effect of method, location and duration on the dependent variables, which were the proportional data for each taxon (mammals, birds, reptiles and amphibians) and often contained many zeros. Indeed, due to the very high number of zeros in the reptile (*n* = 28 zeros) and amphibian data (*n* = 54 zeros), zero‐inflated beta regressions were used for these taxa. A lack of suitable frequentist R packages led to Bayesian analysis being most suited to this proportional dataset. Whilst zero‐inflation models are an option in many cases, only 13 of the data points (14.8%) for fish were above zero, and just two studies indicated that more than 3% of overall diet was fish. These fish data were therefore not analysed. One single model was used for all four prey groups, i.e., with four dependent variables simultaneously. For reptiles and amphibians, the model specified zero‐inflation, whilst for mammal and bird datasets, zero‐inflation was not apparent, and these taxa were therefore analysed using simple beta regression, still in the same model. As birds were not detected in two studies, and mammals were not recorded in a further study (leaving a single zero datapoint for mammals and two for birds), these data were initially not suitable for simple beta regression (as data cannot contain zeros or ones). We therefore transformed them using the following equation, where N is the sample size and Y_i_ represents individual prey proportions (Zuur and Ieno 2016):

$$\left(Y_{i}\times \left(N-1\right)+0.5\right)/N$$

When building the final model, goodness of fit was determined by plotting the posterior mean of *Y* against observed *Y*. Variables with a stronger impact were selected for inclusion in the final model (methods, location and duration were included, cat type was excluded as discussed). We used 20,000 iterations with a burn‐in of 5000 iterations (thinning rate of five and three chains) for the final models, hence the total number of iterations used was 3 × (20,000–5000)/5 = 9000 iterations. Variables were relevelled and the models were set to run once with each variable as a reference, such that we used return studies, capture studies and consumption study data once as the reference variable for each taxon (along with relevelling location in the same way). The results presented here refer to ‘statistical importance’ rather than significance, as this terminology is considered most appropriate when working within a Bayesian framework. An important effect was observed where the credible interval of the posterior mean did not cross zero, for which, a confidence threshold of 95% was used.

A subset of studies (*n* = 18, all from mainland Europe) was found to present sufficiently detailed data, such that taxa could be further divided (e.g., insectivores could be examined separately from rodents). These studies were selected for frequentist comparison using Wilcoxon Rank Sum tests, examining potential differences in prey proportions between consumption and return methods. Not all consumption studies compared here concerned feral cats, since two return studies reported details of prey eaten or part‐eaten (Borkenhagen 1978; Lockwood et al. 2025). For this series of tests, prey were grouped into the following categories:

1. Rodents, including mice, rats and voles
2. Insectivores, including shrews, moles and bats
3. Medium mammals, including rabbits, hares and mustelids
4. Birds, including eggs
5. Reptiles
6. Amphibians
7. Fish

Whilst datasets included in this smaller, more detailed analysis were intentionally restricted to mainland Europe in an attempt to limit effects of varying prey availability between areas, we recognise that Europe is a vast area with a wide variety of habitats and climatic differences which may affect prey proportions available. Therefore, we additionally used a small number of paired t‐tests (*n* = 4) on studies which monitored both eaten and returned prey within the same area (Borkenhagen 1978; Krauze‐Gryz et al. 2012; Piontek et al. 2021; Lockwood et al. 2025).

All analyses were carried out using R version 4.0.2 and R Studio version 1.0.153 (R Studio Team 2020; R Core Team 2022). The packages ‘R2jags’ (Su and Yajima 2020) and ‘zoib’ (Liu and Kong 2019) were used for Bayesian analysis, whilst frequentist testing was conducted under base R (R Core Team 2022). Plots were created using package ‘ggplot2’ (Wickham 2016) and base R (R Core Team 2022).

## 3 Results

### 3.1 Main Meta‐Analysis

After applying the inclusion and exclusion criteria, 80 articles were selected for inclusion in the meta‐analysis (Figure 1). However, 10 articles used multiple methods or studied more than one population of cats (Nilsson 1940; Eberhard 1954; Fitzgerald et al. 1991; Brickner‐Braun et al. 2007; Peck et al. 2008; Krauze‐Gryz et al. 2012; Lanszki et al. 2015; Piontek et al. 2021; Lockwood 2024) and therefore, this search yielded a total of 90 datasets from 80 published studies (Figure 1, Table S2). Data were compiled from across the globe, including studies from 20 countries, with the earliest dating back to 1936. In 73 of these studies (81%), mammals made up at least 50% of the prey, with an overall mean of 63.8% (Table S2). In two thirds of the studies (*n* = 62, 68.9%), birds were the second most represented prey (after mammals), with an overall mean proportion of 19.3%. After the removal of two data points which did not specify proportions for reptiles and amphibians (only ‘herptiles’), the compiled research for formal analyses included 33 prey return datasets, 6 video studies and 49 consumption datasets.

**FIGURE 2:**
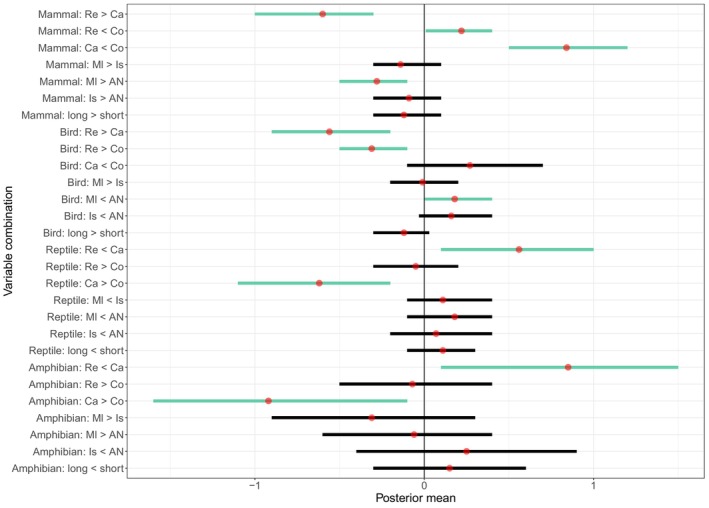
The posterior mean (red points) and credible intervals (horizontal lines) for each variable combination. Where credible intervals cross zero, there is no ‘statistically important’ effect (black lines). Green lines indicate important results, and the direction of the effect is shown on the y axis (< or >), even if this was not considered statistically important because the 95% credible intervals spanned zero. AN, Australia and New Zealand; Ca, caught; Co, consumed; Is, island; L, long; Ml, mainland; Re, returned; S, short.

**FIGURE 3:**
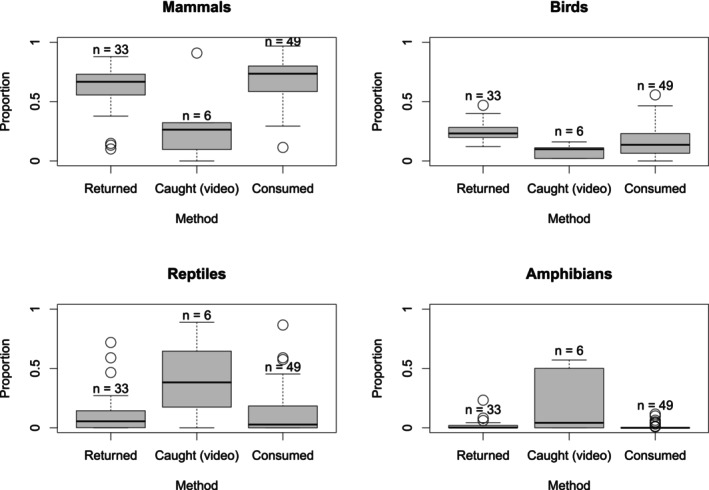
The effect of the method used to determine predation on the estimated proportion of total prey returned, consumed and caught for four main prey groups. Returned = questionnaire studies, caught = video studies, consumed = scat and stomach studies. Sample size (the number of studies) is included above each box. Statistically important differences were found for mammals: Between all method combinations; for birds: Between returned studies and all other methods; for reptiles: Between video capture studies and all other methods and for amphibians: Between video capture studies and all other methods.

For all taxa, the methods used in studies were statistically important (Table 1, Figures 2 and 3). Mammal species occurred in lower proportions in video studies than in those using other methods, and they were proportionally consumed more (i.e., mostly by feral cats) than returned (i.e., by owned cats) (Table 1 and Figure 2). Birds made up a greater proportion of species reported in return studies than those utilising other methods. In contrast to mammals, a far higher proportion of reptiles was reported in the six video studies, and there were statistically important differences between these studies and those using other methods, i.e., return and consumption. Similarly to reptiles, a higher proportion of amphibians was caught (as seen on video footage) than returned in questionnaire or consumption studies (Table 1 and Figure 2).

**TABLE 1:** Beta‐regression outputs, described by variables tested.

Taxon	Variable	*p* Mean	SE	2.5%	97.5%
Mammals	Methods: Re > Ca	**−0.60**	**0.003**	**−1.0**	**−0.3**
	Re < Co	**0.22**	**0.001**	**0.01**	**0.4**
	Ca < Co	**0.84**	**0.003**	**0.5**	**1.2**
	Location: Ml = Is	−0.14	0.001	−0.3	0.1
	Ml > AN	**−0.28**	**0.001**	**−0.5**	**−0.1**
	Is = AN	−0.09	0.001	−0.3	0.1
	Duration: L = S	−0.12	0.001	−0.3	0.1
Birds	Methods: Re > Ca	**−0.56**	**0.003**	**−0.9**	**−0.2**
	Re > Co	**−0.31**	**0.001**	**−0.5**	**−0.1**
	Ca = Co	0.27	0.003	−0.1	0.7
	Location: Ml = Is	−0.01	0.001	−0.2	0.2
	Ml < AN	**0.18**	**0.001**	**0.003**	**0.4**
	Is = AN	0.16	0.001	−0.03	0.4
	Duration: L = S	−0.12	0.001	−0.3	0.03
Reptiles	Methods: Re < Ca	**0.56**	**0.003**	**0.1**	**1.0**
	Re = Co	−0.05	0.002	−0.3	0.2
	Ca > Co	**−0.62**	**0.003**	**−1.1**	**−0.2**
	Location: Ml = Is	0.11	0.002	−0.1	0.4
	Ml = AN	0.18	0.002	−0.1	0.4
	Is = AN	0.07	0.002	−0.2	0.4
	Duration: L = S	0.11	0.001	−0.1	0.3
Amphibians	Methods: Re < Ca	**0.85**	**0.005**	**0.1**	**1.5**
	Re = Co	−0.07	0.003	−0.5	0.4
	Ca > Co	**−0.92**	**0.006**	**−1.6**	**−0.1**
	Location: Ml = Is	−0.31	0.004	−0.9	0.3
	Ml = AN	−0.06	0.003	−0.6	0.4
	Is = AN	0.25	0.004	−0.4	0.9
	Duration: L = S	0.15	0.003	−0.3	0.6

*Note:* Posterior mean (*p* Mean), time‐series standard error (SE) and the 2.5% and 97.5% credible intervals are given. Where credible intervals do not cross zero, a statistically important result is observed. Statistically important values are shown in bold text. The data structure was re‐levelled and re‐run with different reference variables. For example, the first line of results shows returned‐caught, where return survey data were used as the reference. Symbols (<, >, =) are used to indicate the direction of each result. The full model was as follows: Mammals|Birds|Reptiles|Amphibians ~ Methods + Location + Duration.

Abbreviations: AN, Australia and New Zealand; Ca, caught; Co, consumed; Is, island; L, long; Ml, mainland; Re, returned; S, short.

Lower proportions of mammals were recorded in Australasian studies compared to those in mainland environments elsewhere in the world (Table 1 and Figure 2). However, the opposite was true of bird species reported, with greater proportions occurring in the records for Australasia. For herpetofauna, location did not have a statistically important impact on prey proportions. Similarly, the taxon proportions reported did not differ depending on duration (up to a year or at least 12 months). The general patterns seen in the data show that longer studies produce higher proportions of mammals and birds, but lower proportions of reptiles and amphibians, although these differences were not statistically important here.

### 3.2 Comparing Returned and Consumed Prey

**FIGURE 4:**
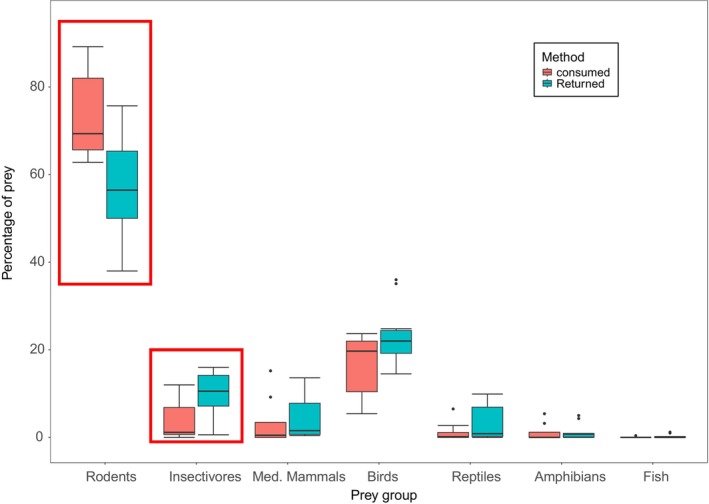
Comparison of prey proportions reported by studies of consumed (red) and returned (blue) prey in mainland Europe. For all prey groups, samples sizes were *n* = 8 consumption studies and *n* = 10 return studies. Mammals are divided into rodents, insectivores and medium mammals (med. mammals, including lagomorphs and mustelids). Significant results are shown within a red box.

The subset of studies with more detailed information on prey type (*n* = 8 consumption data, *n* = 10 return data, Table S3) found that a significantly higher proportion of rodents were eaten than returned (Wilcoxon rank sum test: *W*_8,10_ = 67, *p* = 0.02, Table 2, Figure 4). In contrast, a significantly higher proportion of insectivores were returned than eaten (*W*_8,10_ = 15.5, *p* = 0.03, Table 2, Figure 4). Birds showed a similar trend as insectivores, with a higher proportion returned than eaten, but this was not statistically significant (*W*_8,10_ = 23, *p* = 0.15, Table 2, Figure 4). For studies suitable for paired comparison (*n* = 4), gathering both eaten and returned data in the same location, insectivores were again found to be significantly higher in return data (*t* = −3.2, df = 3, *p* = 0.03, see Table S4), although the patterns for rodents and birds were less clear (Table S4).

**TABLE 2:** Test outputs for a series of Wilcoxon rank sum tests (one for each prey group), examining differences between consumed and returned prey in mainland Europe.

Prey group	*n* (Consumed, returned)	*W*	*p*
Rodents	8, 10	67	0.016*
Insectivores	8, 10	15.5	0.033*
Medium mammals	8, 10	25.5	0.210
Birds	8, 10	23	0.146
Reptiles	8, 10	25	0.189
Amphibians	8, 10	29.5	0.352
Fish	8, 10	32.5	0.393

*Note:* Mammals are divided into rodents, insectivores and ‘medium mammals’ including lagomorphs and mustelids. Significant results are marked with an *. Details of studies used can be found in Table S3.

## 4 Discussion

### 4.1 Data Collection Methods Used

As it has previously been suggested (Lepczyk et al. 2023), here, an important difference was found between prey proportion data gathered using different dietary methods, supporting the first hypothesis. Mammals and birds were reported more frequently in return and consumption data than caught using cameras, whilst opposite patterns were seen for herpetofauna. Similarly, in South Australia, Woinarski et al. (2018), analysing stomach contents, found reptiles to be the most depredated group (by feral cats) whilst McGregor et al. (2015), using video cameras in the same country (although in Western Australia), found that amphibians constituted the highest proportion of captures for feral cats. However, return studies, again in Australia, show mammals to be the top returned taxon (Barratt 1997; Calver et al. 2007; Hall et al. 2015). Of these Australian studies, those concerning feral cats took place relatively far from substantial human settlements (McGregor et al. 2015; Woinarski et al. 2018). However, return surveys are generally in more built‐up areas, since they study the habits of owned cats. Therefore, this difference between most reported prey groups could, in part, be due to an urban–rural difference in prey availability.

Overall, differences were found to exist between all method combinations for mammalian prey, with a lower proportion being recorded using collar‐mounted video cameras than any other method. Camera studies are limited by the short battery life of the devices, meaning that each cat is only monitored for a brief period. For example, whilst Loyd et al. (2013) studied cats for over 12 months in total, each cat collected up to 10 days of data with cameras having a battery life of up to 12 h. Alternatively, the reduced proportions of mammals observed in video studies may suggest that cat‐cameras in some way impede the cats' ability to hunt mammals more than for other taxa, although this is unlikely as they are best suited to capturing ground‐dwelling prey (Leyhausen 1979) and the cameras are generally of small proportions and weight (Bruce et al. 2019; Seymour et al. 2020). The heavier units (90 g, used by Loyd et al. 2013) are more likely to negatively affect hunting ability than the lighter units (32 g, used by Seymour et al. 2020), especially as the lighter units also have smaller proportions and are not as bulky (Lockwood 2024). It is also possible that some models of video camera used may emit a light (to indicate recording mode), potentially acting as a warning for small mammals at night, reducing predation success. An alternative explanation for the relatively low proportions of mammals in video capture studies may simply be a product of increased herptile captures. Amphibians and reptiles may be harder to carry (thus not appearing as frequently in return surveys) or more prevalent in locations where the video studies were conducted, as the six studies included here monitored predation on four continents, generally in areas of non‐typical prey availability (e.g., protected landscapes, Seymour et al. 2020).

Unlike mammals, birds were reported in higher proportions in owner questionnaires than consumption studies and those using cameras, which may be due to detectability bias. As the evidence of bird capture is more easily visible to cat owners than small mammal remains in many cases, owing to a difference in digestibility (e.g., beaks and feathers, Lockwood et al. 2025), birds are perhaps more accurately represented in return surveys than mammals.

In addition to this difference between video studies and other methods used, a further pattern was found in the mammalian data. In consumption studies, higher proportions of mammals were reported than in those using return surveys. Due to the correlation between cat type and method (i.e., return studies all concerning owned cats and consumption studies being chiefly on feral individuals), this pattern could, in part, reflect differences between feral and owned cat diet, with feral individuals hunting for survival. However, it may be more likely that since feral cats are commonly monitored in more rural landscapes, they have access to higher mammal populations than more urban areas where owned cats are generally surveyed, as similar patterns have been found between urban and rural cats (Kauhala et al. 2015). Of course, return surveys and consumption studies fundamentally measure different things in different cat populations (prey carried home by owned cats and prey eaten by feral cats). Therefore, it is unsurprising that a difference was found here and the precise cause of this pattern remains rather unclear and speculative. More data on the actual consumption of prey by pet domestic cats, such as those of Lanszki et al. (2015) and Piontek et al. (2021), would be important to help disentangle the effect of cat type, differences along the urban–rural gradient and methodological approach or measurement used, i.e., capture, consumption and prey carried home.

The differences in prey proportions reported for each method strongly and reasonably suggest that taxa are returned, consumed and caught in varying proportions. Indeed, in video capture studies, mammals have been observed to be returned 50% and 66.67% of the time (Loyd et al. 2013; Seymour et al. 2020, respectively). However, in the same studies, reptiles were returned only 7.14% of the time (Loyd et al. 2013) or not at all (of 31 capture events, Seymour et al. 2020). Such camera studies, along with direct observations, can be used to develop factors by which return data can be multiplied, estimating total predation. For example, Kays and DeWan (2004) found that cats return 30% of all prey caught, suggesting 3.3 as a multiplication factor. However, as shown here, a single multiplier is not appropriate for extrapolation, as this can lead to sizeable under‐ and over‐estimates of predation on different taxa. The 3.3× multiplier was calculated using mammal and bird data (Kays and DeWan 2004) and can only reasonably be used to extrapolate from return data in the same proportions. It is therefore not appropriate for use with reptile or amphibian calculations. In addition, since the majority of prey in this instance were mammals (Kays and DeWan 2004), the 3.3× multiplier should also not be used to extrapolate bird‐only data (as has been done in previous works, Baker et al. 2008; Maclean et al. 2008). Indeed, the bird data from Kays and DeWan (2004) show that whilst eight birds were returned home, when conducting direct behavioural observations, no birds were caught by cats. Therefore, using this multiplier can easily lead to over‐estimates of bird captures and the overall impact of cats on local bird populations, since its use suggests that more birds are caught than returned, whilst this was not the case in this example. There currently does not appear to be a reliable multiplier for use with bird calculations, as birds are not as frequently caught as mammals (in collar‐mounted video studies, Loyd et al. 2013; Seymour et al. 2020), and therefore, more extensive research is required to produce a good estimate of the proportion of captures carried home.

### 4.2 Island, Mainland and Australasian Studies

In addition to methods used, it was found here that the location of study should be considered when conducting large‐scale predation extrapolations or estimating the impact of cats, since the proportions of mammals and birds reported were influenced by location (supporting the second hypothesis). Indeed, mammal records from Australia and New Zealand were lower than those in mainland areas elsewhere in the world, which can be explained, in part, by the faunal composition of Australasia, with New Zealand having no native terrestrial mammals (IUCN 2020). Conversely, for birds, higher proportions were reported in Australasia than in other mainland areas, which is also likely a result of prey availability. The differences seen here between continental regions emphasise the importance of repeating such studies around the world and not using the results of Australasian studies to inform conservation measures in Europe or the USA, for example.

### 4.3 Consumed and Returned Prey Analysis

Whilst differences between continents may seem a rather obvious finding, the palatability of different prey categories is a consideration that has, so far, only rarely been considered (Krauze‐Gryz et al. 2012; Lockwood et al. 2025). It has been hypothesised that insectivores are unpalatable to cats (Toner 1956; Turner and Bateson 2000; Krauze‐Gryz et al. 2012; Kauhala et al. 2015), which may explain the significant difference in consumed and returned individuals seen here. In the smaller analysis (paired *t*‐tests, Table S4), only those studies gathering both returned and eaten data in mainland Europe were included, in an attempt to limit the effect of varying prey availability. Despite the very low sample size (*n* = 4 studies), the same pattern between eaten and returned insectivores was observed, with higher proportions appearing in return data. This significant result may become further strengthened with the inclusion of other similar studies if these data were available. Secondary to palatability, this pattern may also be related to the age and experience of cats monitored. Indeed, Kauhala et al. (2015) found that insectivores and reptiles are more commonly brought home by younger cats, suggesting that with age and experience, cats learn that these species are less palatable. As they are more experienced, feral cats (most consumption studies) may predate these groups less frequently, along with older owned cats.

In addition to this pattern in insectivore records, dividing mammals into sub‐groups also found significant differences between eaten and returned rodents, with a higher proportion being consumed. Although sample sizes here were rather low (*n* = 8 consumption data, *n* = 10 returned data), this pattern suggests that rodents are highly palatable, frequently appearing in the diet of feral cats (Fitzgerald et al. 1991; Biró et al. 2005; Bonnaud et al. 2007; Krauze‐Gryz et al. 2012). In contrast to rodents, yet similar to insectivores, birds appear to be more often returned than consumed. Although not significant here, this pattern in the bird data may become significant as it gains more statistical power, given more datapoints. An alternative but not mutually exclusive hypothesis to palatability may be that this result reflects a difference in detectability of prey remains, with bird remains (feathers and beaks) being more easily detected in prey return surveys than mammal remains (where there is often little remaining, Lockwood et al. 2025). It is important that palatability and detectability of prey are considered in future work and also when interpreting existing research, in order to minimise over‐ and under‐representation of different taxonomic groups in total predation estimates.

### 4.4 Limitations

Whilst not recorded here due to inconsistencies of reporting, the habitat and level of urbanisation may have also influenced the proportions of prey observed. For example, Krauze‐Gryz et al. (2017) found that cats in urban areas of Poland kill three times as many birds (per cat) as their rural counterparts, with similar effects seen in Finland (Kauhala et al. 2015) and Australia (Barratt 1997). However, fine‐scale habitat is not always possible to assess, as some studies have been conducted on a large spatial scale, incorporating different levels of urbanisation without providing details of this (e.g., Woods et al. 2003). In order to aid future meta‐analytical studies, it would be helpful for the authors to provide some measure of urbanisation (e.g., human density) at the time the study was conducted, although this might not be possible for large‐scale studies.

Furthermore, the mass of prey was not considered here, as further categorising prey could lead to over‐parameterisation effects where sample sizes are low. Excluding such data is a limitation of the current study, as the mass of prey will likely impact the diversity and number of prey caught (or at least consumed) in a day, having an effect on scat, stomach and gut contents. For example, if a cat consumes an adult rabbit, it is unlikely that much else will be eaten in the same sampling period, leaving a record of 100% mammalian prey for that individual. Not including mass could be a particular concern for studies in more rural habitats where larger‐bodied prey are more available, and those on feral individuals which are reliant on wild prey. However, using biomass as a measure could greatly affect the proportions reported. For example, as invertebrates are generally small‐bodied, yet can be eaten in large numbers, the use of percentage biomass in a study such as this could lead to the potential effects of predation being underestimated. Indeed, Woinarski et al. (2018) found that whilst invertebrates made up the majority of individual prey items (69%), this taxon represented only 7.5% of the total prey biomass. As a valuable dietary measure allowing prey species to be ranked by relative importance within the diet of cats (Nogales et al. 1988), biomass should perhaps be considered and controlled for in future analyses, given detailed prey species data and a large enough dataset to avoid over‐parameterisation.

Invertebrates were excluded from all analyses and proportions were recalculated to adjust for these missing data. Whilst this decision was influenced by inconsistencies in the reporting of this taxonomic group, it is important to recognise that conclusions drawn here do not include these prey. Although some consumption studies report rather high proportions of invertebrates (e.g., 50% of feral cat stomach content data collected on a French island, before adjustment, Peck et al. 2008), it is not clear whether this is higher in feral individuals hunting for survival. Indeed, Woolley et al. (2020) estimated that each feral cat in Australia consumes around 371 invertebrates annually. However, the conservation status of invertebrate prey has not yet been fully assessed and, therefore, the impact cannot be understood. Of the 70 invertebrate species listed in a review by Bonnaud et al. (2011), only one, the emperor dragonfly (*Anax imperator*), had been assigned an IUCN status, Least Concern. The populations of the remaining 69 species had not been assessed (Bonnaud et al. 2011). Owned pet cats are also known to catch invertebrates (Loyd et al. 2013) and figures for such species can be relatively high when recorded consistently (47% of prey returned home before adjustment, Gillies and Clout 2003), although most studies reported zero invertebrates.

When exploring the data in more detail, further dividing mammals into three separate groups, only mainland European studies were included to minimise differences in prey availability between studies. Although prey species availability is likely not the same between sites (as this can differ even on a fine scale), taxon availability may be rather more constant between mainland European sites. Despite the potential variability in prey availability, palatability remains areas of interest, since estimates of total predation (including returned, discarded and eaten prey) may be improved by accounting for proportions eaten or otherwise not detected.

It should also be considered that most of the cat populations in the ‘consumption’ category monitored in the smaller, more detailed analysis were feral individuals (*n* = 4), whereas all of those in the ‘return’ category were owned cats (see Table S1 for descriptions). The results presented here may, therefore, reflect this difference between feral and owned individuals, with owned cats potentially depredating a greater proportion of rodents and lower proportions of insectivores and birds than feral individuals. Since feral cat studies are generally conducted in more rural locations, and those on owned cats in more urban or suburban areas, this difference may also be a reflection of a difference in prey availability across an urbanisation gradient. Despite this potential urban–rural difference, we provide details of very small (*n* = 4) paired t‐tests (in Table S4), which show that where location is constant between consumed and returned data, similar patterns are apparent, particularly for insectivores.

### 4.5 Conclusions

Both the location of studies and the methods used to gather dietary data for cats can quite clearly affect the proportions of prey taxa reported. It is, therefore, important that sweeping statements regarding predation rates are not made based on data gathered using different methods and studies in different locations, as this may not be representative of other areas or populations. For example, studies of the prey caught in New Zealand cannot be used to assess the impact of cats in the UK. It is clear that more research is needed using video around the world and on both feral and owned cats, improving the reliability and applicability of these data to wider cat populations, as there was such large variability in the results of the few studies found here. The resulting video data from such new research can then be used to generate new multipliers for estimating total predation rates from return data, taking into account differences between taxonomic groups. Palatability and detectability of prey should also be considered in future research, or at least monitored allowing for more detailed future analysis.

In the interest of replicability and further meta‐analytical study, studies of cat diet should ideally last for at least a year, covering a large area with a large sample of cats. Recording the human density in the area of study (or some other measure of urbanisation) in future would allow for an even more comprehensive meta‐analytical review. Improving the methods used will ultimately improve the quality of data produced, which is fundamental for the future conservation of prey species of concern around the world, as understanding exactly what is being killed (or otherwise affected) by cats is the first step in planning conservation actions. As the technology available develops, higher capacity batteries may be used in small, lightweight camera devices, thus improving the volume of data gathered. A larger sample size in video studies would vastly improve the reliability of results, and patterns of taxa eaten, discarded and returned home would emerge more strongly.

Of course, the present study does not assess the impact of cats, as it only looks at prey proportions with no measure of magnitude. Whilst impact is surely an important area of study, in order to produce reliable estimates, one must first estimate the number of prey caught as accurately as possible. Therefore, despite not directly investigating the effects of non‐native cat populations, this study does have implications for extrapolation multipliers and, therefore, impact estimates.

## Author Contributions

**Hannah L. Lockwood:** conceptualization (equal), data curation (lead), formal analysis (equal), methodology (equal), writing – original draft (lead), writing – review and editing (equal). **Maren Huck:** conceptualization (equal), formal analysis (equal), methodology (equal), supervision (lead), writing – review and editing (equal).

## Conflicts of Interest

The authors declare no conflicts of interest.

## Supporting information


Data S1.

## Data Availability

Data are all available within the manuscript and Data S1.
