# Supplementary material for: A Global Systematic Review and Meta‐Analysis of Methods Used to Evaluate Predation and Diet of Domestic Cats (Felis catus)
Source: Ecol Evol. 2025 Apr 25;15(4):e71349. doi: 10.1002/ece3.71349 (PMC12022773; doi:10.1002/ece3.71349)
Supplement: Supplementary file 1 — Data S1. [file ECE3-15-e71349-s001.docx]

Supporting Information

Table S1. Descriptions of the feral and owned categories used here, compared with those described by Lepczyk and Calver (2022).

| **Category used here** | **Category used by Lepczyk and Calver (2022)** | **Description** |
| --- | --- | --- |
| Owned | Owned (indoor-outdoor) | - Partly confined, but given roaming opportunities - Regularly provided with a suitable diet - Population is sustained by human involvement |
| Feral | Stray  (free-roaming, homeless, colony and community cats) | - Largely or entirely reliant on prey to survive - Unowned and free-roaming, but diet may be subsidised by local people |
|  | Feral | - Entirely self-sustaining, often living far from people |

Table S2. Percentage of mammals, birds, reptiles, amphibians, and fish in the diets of cats recorded in 90 individual populations or studies (80 articles) estimating cat predation rates. Islands are classed as pieces of land no larger than 100,000 km^2^. Where there are multiple values, more than one treatment was applied. Australia and New Zealand are denoted as ‘AU/NZ’. Where ‘herpetofauna’ was stated, cells are merged (n=2) and these studies were not included in formal analyses. Dark grey shading denotes a higher percentage, and light grey a lower percentage. Longer studies (over 12 months) are marked with an *.

| **Reference** | **Year** | **Feral/ Owned** | **Methods** | **Country** | **Location** | **12 months +** | **Mammals** | **Birds** | **Reptiles** | **Amphibians** | **Fish** |
| --- | --- | --- | --- | --- | --- | --- | --- | --- | --- | --- | --- |
| Borkenhagen | 1978 | Owned | Questionnaire | DE | Mainland | * | 77.6% | 22.1% | 0.3% | 0.0% | 0.0% |
| Churcher & Lawton | 1987 | Owned | Questionnaire | UK | Mainland | * | 64.4% | 35.8% | 0.0% | 0.0% | 0.0% |
| Mitchell & Beck | 1992 | Owned | Questionnaire | US | Mainland | * | 54.0% | 19.8% | 22.5% | 3.7% | 0.0% |
| Carss | 1995 | Owned | Questionnaire | UK | Mainland | * | 85.0% | 14.7% | 0.2% | 0.0% | 0.0% |
| Barratt | 1997 | Owned | Questionnaire | AU | AU/NZ | * | 65.0% | 27.0% | 6.7% | 1.1% | 0.2% |
| Howes | 2002 | Owned | Questionnaire | UK | Mainland | * | 69.1% | 30.5% | 0.0% | 0.2% | 0.1% |
| Ruxton *et al.* | 2002 | Owned | Questionnaire | UK | Mainland |  | 72.4% | 21.5% | 0.0% | 6.1% | 0.0% |
| Gillies & Clout | 2003 | Owned | Questionnaire | NZ | AU/NZ | * | 59.9% | 25.7% | 14.4% | 0.2% | 0.0% |
| Woods *et al.* | 2003 | Owned | Questionnaire | UK | Mainland |  | 70.4% | 24.2% | 1.0% | 4.2% | 0.2% |
| Kays & DeWan | 2004 | Owned | Questionnaire | US | Mainland |  | 86.4% | 13.6% | 0.0% | 0.0% | 0.0% |
| Baker *et al.* | 2005 | Owned | Questionnaire | UK | Mainland | * | 75.1% | 24.0% | 0.0% | 0.8% | 0.0% |
| Nelson *et al.* | 2005 | Owned | Questionnaire | CH | Mainland |  | 65.0% | 32.3% | 0.6% | 2.0% | 0.1% |
| Brickner-Braun *et al.* | 2007 | Owned | Questionnaire | IL | Mainland | * | 55.6% | 20.1% | 24.4% | 0.0% | 0.0% |
| Calver *et al.* | 2007 | Owned | Questionnaire | AU | AU/NZ |  | 55.4% | 22.0% | 22.6% | | 0.0% |
| Flux | 2007 | Owned | Questionnaire | NZ | AU/NZ | * | 58.2% | 40.0% | 1.6% | 0.2% | 0.0% |
| Baker *et al.* | 2008 | Owned | Questionnaire | UK | Mainland | * | 69.3% | 20.6% | 5.5% | 4.4% | 0.0% |
| Morgan *et al.* | 2009 | Owned | Questionnaire | NZ | AU/NZ | * | 50.2% | 26.2% | 22.6% | 0.6% | 0.3% |
| van Heezik *et al.* | 2010 | Owned | Questionnaire | NZ | AU/NZ | * | 42.7% | 46.9% | 10.1% | 0.2% | 0.0% |
| Tschanz *et al.* | 2011 | Owned | Questionnaire | UK | Mainland |  | 87.9% | 12.1% | 0.0% | 0.0% | 0.0% |
| Krauze-Gryz *et al.* | 2012 | Owned | Questionnaire | PL | Mainland | * | 76.0% | 14.3% | 7.8% | 0.8% | 1.0% |
| Thomas *et al.* | 2012 | Owned | Questionnaire | UK | Mainland | * | 64.7% | 30.5% | 0.5% | 4.4% | 0.0% |
| Hall *et al.* | 2015 | Owned | Questionnaire | AU | AU/NZ |  | 45.3% | 27.6% | 27.1% | 0.0% | 0.0% |
| Kauhala *et al.* | 2015 | Owned | Questionnaire | FI | Mainland |  | 79.2% | 17.8% | 3.0% | | 0.0% |
| 00.00.0McDonald et al. | 2015 | Owned | Questionnaire | UK | Mainland | * | 62.5% | 28.3% | 9.3% | 0.0% | 0.0% |
| Willson *et al.* | 2015 | Owned | Questionnaire | US | Mainland |  | 73.1% | 26.9% | 0.0% | 0.0% | 0.0% |
| Wood *et al.* | 2016 | Owned | Questionnaire | NZ | AU/NZ |  | 66.7% | 33.3% | 0.0% | 0.0% | 0.0% |
| Krauze-Gryz *et al.* | 2017 | Owned | Questionnaire | PL | Mainland | * | 72.7% | 15.5% | 9.7% | 0.9% | 1.2% |
| Mori *et al.* | 2019 | Owned | Questionnaire | IT | Mainland | * | 37.8% | 35.4% | 23.7% | 3.1% | 0.0% |
| Castañeda *et al.* | 2020 | Owned | Questionnaire | FR | Mainland | * | 70.2% | 21.4% | 8.4% | 0.0% | 0.0% |
| Piontek *et al.* | 2021 | Owned | Questionnaire | PL | Mainland | * | 73.2% | 23.2% | 3.6% | 0.0% | 0.0% |
| Geiger *et al.* | 2022 | Owned | Questionnaire | CH | Mainland |  | 68.2% | 22.7% | 9.1% | 0.0% | 0.0% |
| Jensen *et al.* | 2022 | Owned | Questionnaire | US | Mainland |  | 13.3% | 13.3% | 71.9% | 1.5% | 0.0% |
| Mella-Méndez *et al.* | 2022 | Owned | Questionnaire | MX | Mainland |  | 10.1% | 20.1% | 46.6% | 23.3% | 0.0% |
| Lockwood *et al.* | 2025 | Owned | Questionnaire | UK | Mainland | * | 83.2% | 16.0% | 0.1% | 0.6% | 0.0% |
| Panchana *et al.* | 2025 | Owned | Questionnaire | EC | Mainland |  | 14.8% | 18.0% | 59.0% | 8.2% | 0.0% |
| Loyd et al. | 2013 | Owned | Video | US | Mainland | * | 32.2% | 16.1% | 45.2% | 6.4% | 0.0% |
| McGregor et al. | 2015 | Feral | Video | AU | AU/NZ | * | 21.5% | 10.7% | 17.5% | 50.1% | 0.0% |
| Hernandez *et al.* | 2018 | Feral | Video | US | Mainland |  | 9.7% | 2.1% | 31.3% | 57.1% | 0.0% |
| Bruce *et al.* | 2019 | Owned | Video | NZ | AU/NZ |  | 0.0% | 11.1% | 88.9% | 0.0% | 0.0% |
| Seymour *et al.* | 2020 | Owned | Video | ZA | Mainland |  | 31.3% | 2.1% | 64.6% | 2.1% | 0.0% |
| Lockwood | 2024 | Owned | Video | UK | Mainland | * | 90.9% | 9.1% | 0.0% | 0.0% | 0.0% |
| Nilsson | 1940 | feral | Scat | US | Mainland | * | 66.1% | 33.9% | 0.0% | 0.0% | 0.0% |
| Eberhard | 1954 | Feral | Scat | US | Mainland | * | 73.5% | 26.5% | 0.0% | 0.0% | 0.0% |
| Karl & Best | 1982 | Feral | Scat | NZ | AU/NZ | * | 59.9% | 15.9% | 24.3% | 0.0% | 0.0% |
| Apps | 1983 | Feral | Scat | ZA | Island | * | 62.4% | 37.6% | 0.0% | 0.0% | 0.0% |
| Nogales *et al.* | 1988 | Feral | Scat | ES | Island |  | 58.5% | 4.7% | 36.6% | 0.0% | 0.0% |
| Fitzgerald *et al.* | 1991 | Feral | Scat | NZ | AU/NZ |  | 86.9% | 13.1% | 0.0% | 0.0% | 0.0% |
| Weber & Dailly | 1998 | Feral | Scat | CH | Mainland | * | 94.6% | 5.4% | 0.0% | 0.0% | 0.0% |
| Smucker *et al.* | 2000 | Feral | Scat | US | Island | * | 68.3% | 31.7% | 0.0% | 0.0% | 0.0% |
| Medina *et al.* | 2006 | Feral | Scat | ES | Island | * | 67.3% | 5.6% | 27.1% | 0.0% | 0.0% |
| Bonnaud *et al.* | 2007 | Feral | Scat | FR | Island | * | 91.2% | 6.9% | 1.9% | 0.0% | 0.0% |
| Campos *et al.* | 2007 | Feral | Scat | BR | Mainland | * | 55.9% | 34.9% | 4.6% | 0.0% | 4.6% |
| Phillips *et al.* | 2007 | Feral | Scat | US | Island | * | 66.1% | 4.7% | 29.2% | 0.0% | 0.0% |
| Matias & Catry | 2008 | Feral | Scat | UK | Island |  | 77.8% | 22.2% | 0.0% | 0.0% | 0.0% |
| Medina *et al.* | 2008 | Feral | Scat | ES | Island |  | 78.7% | 5.9% | 15.6% | 0.0% | 0.0% |
| Peck et al. | 2008 | Feral | Scat | FR | Island |  | 75.5% | 21.7% | 2.8% | 0.0% | 0.0% |
| Faulquier *et al.* | 2009 | Feral | Scat | FR | Island | * | 53.5% | 46.5% | 0.0% | 0.0% | 0.0% |
| Millán | 2010 | Feral | Scat | ES | Island | * | 89.2% | 9.7% | 1.1% | 0.0% | 0.0% |
| Krauze-Gryz *et al.* | 2012 | Feral | Scat | PL | Mainland | * | 74.2% | 17.4% | 3.4% | 5.0% | 0.0% |
| Ferreira *et al.* | 2014 | Feral | Scat | BR | Mainland | * | 76.6% | 22.1% | 0.0% | 1.3% | 0.0% |
| Hervías *et al.* | 2014 | Feral | Scat | ES | Island | * | 73.1% | 26.9% | 0.0% | 0.0% | 0.0% |
| Kitts-Morgan | 2015 | Feral | Scat | US | Mainland | * | 81.5% | 18.5% | 0.0% | 0.0% | 0.0% |
| Lanszki *et al.* | 2015 | Feral | Scat | HR | Island | * | 74.7% | 5.0% | 18.4% | 0.0% | 1.9% |
| Lanszki *et al.* | 2015 | Owned | Scat | HR | Island | * | 58.1% | 18.2% | 3.4% | 0.0% | 20.2% |
| Shionosaki *et al.* | 2015 | Feral | Scat | JP | Island | * | 96.8% | 2.5% | 0.7% | 0.0% | 0.0% |
| Carrión & Valle | 2018 | Feral | Scat | EC | Island |  | 42.5% | 0.0% | 57.5% | 0.0% | 0.0% |
| Medina *et al.* | 2021 | Feral | Scat | CV | Island | * | 11.4% | 2.0% | 86.6% | 0.0% | 0.0% |
| Piontek *et al.* | 2021 | Owned | Scat | PL | Mainland | * | 76.9% | 23.1% | 0.0% | 0.0% | 0.0% |
| Hortelano-Moncada *et al.* | 2024 | Feral | Scat | MX | Mainland | * | 82.5% | 0.0% | 17.5% | 0.0% | 0.0% |
| Errington | 1936 | Feral | Stomach | US | Mainland | * | 86.4% | 13.6% | 0.0% | 0.0% | 0.0% |
| Nilsson | 1940 | Feral | Stomach | US | Mainland | * | 72.7% | 26.3% | 0.0% | 1.0% | 0.0% |
| McMurry & Sperry | 1941 | Feral | Stomach | US | Mainland | * | 90.2% | 6.5% | 3.3% | 0.0% | 0.0% |
| Hubbs | 1951 | Feral | Stomach | US | Mainland | * | 76.5% | 19.5% | 2.6% | 0.0% | 1.4% |
| Parmalee | 1953 | Feral | Stomach | US | Mainland | * | 75.7% | 10.8% | 13.5% | 0.0% | 0.0% |
| Eberhard | 1954 | Feral | Stomach | US | Mainland | * | 73.3% | 26.7% | 0.0% | 0.0% | 0.0% |
| Jones | 1977 | Feral | Stomach | NZ | AU/NZ | * | 58.5% | 41.5% | 0.0% | 0.0% | 0.0% |
| Bloomer & Bester | 1990 | Feral | Stomach | ZA | Island | * | 44.3% | 55.7% | 0.0% | 0.0% | 0.0% |
| Fitzgerald *et al.* | 1991 | Feral | Stomach | NZ | AU/NZ |  | 86.7% | 13.3% | 0.0% | 0.0% | 0.0% |
| Biró *et al.* | 2005 | Feral | Stomach | HU | Mainland | * | 87.8% | 11.2% | 0.6% | 0.0% | 0.4% |
| Brickner-Braun *et al.* | 2007 | Feral | Stomach | IL | Mainland | * | 75.0% | 6.0% | 9.0% | 10.0% | 0.0% |
| Peck *et al.* | 2008 | Feral | Stomach | FR | Island |  | 57.6% | 30.8% | 11.6% | 0.0% | 0.0% |
| Kutt | 2011 | Feral | Stomach | AU | AU/NZ | * | 36.8% | 13.6% | 45.3% | 4.3% | 0.0% |
| Krauze-Gryz *et al.* | 2012 | Feral | Stomach | PL | Mainland | * | 82.2% | 8.0% | 6.4% | 3.2% | 0.0% |
| Yip *et al.* | 2014 | Feral | Stomach | AU | AU/NZ |  | 49.0% | 12.6% | 24.2% | 11.6% | 2.7% |
| Yip *et al.* | 2015 | Feral | Stomach | AU | AU/NZ |  | 52.2% | 12.2% | 27.9% | 6.4% | 1.3% |
| Read et al. | 2018 | Feral | Stomach | AU | AU/NZ | * | 51.2% | 17.9% | 30.9% | 0.0% | 0.0% |
| Woinarski *et al.* | 2018 | Feral | Stomach | AU | AU/NZ | * | 29.4% | 10.7% | 59.0% | 0.9% | 0.0% |
| Piontek *et al.* | 2021 | Owned | Stomach | PL | Mainland | * | 80.0% | 20.0% | 0.0% | 0.0% | 0.0% |
| Hodgens *et al.* | 2022 | Feral | Stomach | AU | AU/NZ |  | 77.7% | 18.3% | 4.0% | 0.0% | 0.0% |
| Read *et al.* | 2024 | Feral | Stomach | AU | AU/NZ | * | 64.1% | 6.4% | 28.7% | 0.8% | 0.0% |

Table S3. Percentage of different prey groups reported as being consumed or returned in different studies. Mammals are divided into rodents, insectivores, and ‘medium mammals’ including lagomorphs and mustelids. The two-letter country code is given as a location for each study.

| **Reference** | **Year** | **Consumed or**  **returned** | **Location** | **Percentage of vertebrate prey** | | | | | | |
| --- | --- | --- | --- | --- | --- | --- | --- | --- | --- | --- |
|  |  |  |  | **Rodents** | **Insectivores** | **Medium**  **Mammals** | **Birds** | **Reptiles** | **Amphibians** | **Fish** |
| Borkenhagen | 1978 | Consumed^1^ | DE | 62.8 | 0.5 | 15.2 | 21.5 | 0 | 0 | 0 |
| Weber & Daily | 1998 | Consumed | CH | 89.2 | 5.4 | 0 | 5.4 | 0 | 0 | 0 |
| Biró *et al.* | 2005 | Consumed | HU | 86.3 | 0 | 1.5 | 11.2 | 0.6 | 0 | 0.4 |
| Krauze- Gryz *et al.* | 2012 | Consumed | PL | 70.7 | 0.7 | 1 | 19.4 | 2.7 | 5.4 | 0 |
| Krauze- Gryz *et al.* | 2012 | Consumed | PL | 80.6 | 1.6 | 0 | 8.1 | 6.5 | 3.2 | 0 |
| Piontek *et al.* | 2021 | Consumed | PL | 65.2 | 11.1 | 0 | 23.7 | 0 | 0 | 0 |
| Piontek *et al.* | 2021 | Consumed | PL | 68 | 12 | 0 | 20 | 0 | 0 | 0 |
| Lockwood *et al.* | 2025 | Consumed^1^ | UK | 65.8 | 0.7 | 9.2 | 23.4 | 0.3 | 0.5 | 0 |
| Borkenhagen | 1978 | Returned | DE | 48.4 | 15.6 | 13.6 | 22.1 | 0.3 | 0 | 0 |
| Churcher & Lawton | 1987 | Returned | UK | 38 | 16 | 11 | 36 | 0 | 0 | 0 |
| Woods *et al.* | 2003 | Returned | UK | 46.6 | 13.8 | 9.2 | 24.8 | 1.1 | 4.3 | 0.2 |
| Baker *et al.* | 2005 | Returned | UK | 75.7 | 0.6 | 1.2 | 21.6 | 0 | 0.9 | 0 |
| Thomas *et al.* | 2012 | Returned | UK | 54.9 | 4 | 0.4 | 35.1 | 0.6 | 5 | 0 |
| Krauze- Gryz *et al.* | 2012 | Returned | PL | 66.1 | 9.1 | 0.5 | 14.5 | 8 | 0.8 | 1 |
| Krauze- Gryz *et al.* | 2017 | Returned | PL | 63.1 | 8.8 | 0.4 | 15.8 | 9.9 | 0.9 | 1.2 |
| Piontek *et al.* | 2021 | Returned | PL | 57.7 | 14.3 | 1.2 | 23.2 | 3.6 | 0 | 0 |
| Castañeda *et al.* | 2023 | Returned | FR | 55.2 | 12 | 1.9 | 21.9 | 8.6 | 0.3 | 0 |
| Lockwood *et al.* | 2025 | Returned | UK | 70.6 | 6.6 | 3.6 | 18.4 | 0.2 | 0.7 | 0 |

^1^ Study used return methods, but gave details of prey returned and eaten or returned part-eaten

Table S4. Test outputs for a series of paired t-tests (for rodents, insectivores, and birds), examining differences between consumed and returned prey in mainland Europe. Pairs share the same study location. Significant results are marked with an *. Studies included were Borkenhagen (1978); Krauze-Gryz *et al.* (2012); Piontek *et al.* (2021); and Lockwood *et al.* (2025).

| **Prey group** | **DF** | ***t*** | ***P*** |
| --- | --- | --- | --- |
| Rodents | 3 | 1.5 | 0.116 |
| Insectivores | 3 | -3.2 | 0.025* |
| Birds | 3 | 1.5 | 0.120 |

References

Apps, P. (1983) Aspects of the ecology of feral cats on Dassen Island, South Africa. *South African Journal of Zoology,* **18**393-399.

Baker, P.J., Bentley, A.J., Ansell, R.J. & Harris, S. (2005) Impact of predation by domestic cats *Felis catus* in an urban area. *Mammal Review,* **35**(3-4)**,** 302-312.

Baker, P.J., Molony, S.E., Stone, E., Cuthill, I.C. & Harris, S. (2008) Cats about town: is predation by free-ranging pet cats *Felis catus* likely to affect urban bird populations? *Ibis,* **150**86-99.

Barratt, D.G. (1997) Predation by house cats, *Felis catus* (L), in Canberra, Australia .1. Prey composition and preference. *Wildlife Research,* **24**(3)**,** 263-277.

Biro, Z., Lanszki, J., Szemethy, L., Heltai, M. & Randi, E. (2005) Feeding habits of feral domestic cats (*Felis catus*), wild cats (*Felis silvestris*) and their hybrids: trophic niche overlap among cat groups in Hungary. *Journal of Zoology,* **266**187-196.

Biró, Z., Lanszki, J., Szemethy, L., Heltai, M. & Randi, E. (2005) Feeding habits of feral domestic cats (*Felis catus*), wild cats (*Felis silvestris*) and their hybrids: trophic niche overlap among cat groups in Hungary. *Journal of Zoology,* **266**(2)**,** 187-196.

Bloomer, J.P. & Bester, M.N. (1990) Diet of a declining feral cat Felis catus population on Marion Island. *South African Journal of Wildlife Research,* **20**(1).

Bonnaud, E., Bourgeois, K., Vidal, E., Kayser, Y., Tranchant, Y. & Legrand, J. (2007) Feeding ecology of a feral cat population on a small Mediterranean island. *Journal of Mammalogy,* **88**(4)**,** 1074-1081.

Borkenhagen, P. (1978) Von Hauskatzen (*Felis sylvestris f. catus* L., 1758) eingetragene Beute. *Zeitschrift für Jagdwissenschaft,* **24**(1)**,** 27-33.

Brickner-Braun, I., Geffen, E. & Yom-Tov, Y. (2007) The Domestic Cat as a Predator of Israeli Wildlife. *Israel Journal of Ecology & Evolution,* **53**(2)**,** 129-142.

Bruce, S.J., Zito, S., Gates, M.C., Aguilar, G., Walker, J.K., Goldwater, N. & Dale, A. (2019) Predation and risk behaviors of free-roaming owned cats in Auckland, New Zealand via the use of animal-borne cameras. *Frontiers in Veterinary Science,* **6**(205)**,** 1-12.

Calver, M., Thomas, S., Bradley, S. & McCutcheon, H. (2007) Reducing the rate of predation on wildlife by pet cats: The efficacy and practicability of collar-mounted pounce protectors. *Biological Conservation,* **137**(3)**,** 341-348.

Campos, C., Esteves, C., Ferraz, M., Crawshaw, P., Verdade, L. & Jr, P. (2007) Diet of free-ranging cats and dogs in a suburban and rural environment, south-eastern Brazil. *Journal of Zoology,* **273**14-20.

Carrion, P.L. & Valle, C.A. (2018) The diet of introduced cats on San Cristobal Island, Galapagos: cat feces as a proxy for cat predation. *Mammalian Biology,* **90**74-77.

Carss, D.N. (1995) Prey brought home by two cats (*Felis catus*) in northern Scotland. *Journal of Zoology,* **237**(4)**,** 678-686.

Castañeda, I., Zarzoso-Lacoste, D. & Bonnaud, E. (2020) Feeding behaviour of red fox and domestic cat populations in suburban areas in the south of Paris. *Urban Ecosystems,* **23**.

Churcher, P. & Lawton, J. (1987) Predation by domestic cats in an English village. *Journal of Zoology,* **212**439-455.

Eberhard, T. (1954) Food habits of Pennsylvania house cats. *The Journal of Wildlife Management,* **18**(2)**,** 284-286.

Errington, P.L. (1936) Notes on food habits of Southern Wisconsin house cats. *Journal of Mammalogy,* **17**(1)**,** 64-65.

Faulquier, L., Fontaine, R., Vidal, E., Salamolard, M. & Le Corre, M. (2009) Feral Cats Felis catus Threaten the Endangered Endemic Barau's Petrel Pterodroma baraui at Reunion Island (Western Indian Ocean). *Waterbirds,* **32**(2)**,** 330-336.

Ferreira, G.A., Nakano-Oliveira, E. & Genaro, G. (2014) Domestic cat predation on Neotropical species in an insular Atlantic Forest remnant in southeastern Brazil. *Wildlife Biology,* **20**(3)**,** 167-175.

Fitzgerald, B.M., Karl, B.J. & Veitch, C.R. (1991) The diet of feral cats (*Felis catus*) on Raoul Island, Kermadec Group. *New Zealand Journal of Ecology,* **15**(2)**,** 123-129.

Flux, J.E.C. (2007) Seventeen years of predation by one suburban cat in New Zealand. *New Zealand Journal of Zoology,* **34**(4)**,** 289-296.

Geiger, M., Kistler, C., Mattmann, P., Jenni, L., Hegglin, D. & Bontadina, F. (2022) Colorful Collar-Covers and Bells Reduce Wildlife Predation by Domestic Cats in a Continental European Setting. *Frontiers in Ecology and Evolution,* **10**.

Gillies, C. & Clout, M. (2003) The prey of domestic cats (*Felis catus*) in two suburbs of Auckland City, New Zealand. *Journal of Zoology,* **259**309-315.

Hall, C.M., Fontaine, J.B., Bryant, K.A. & Calver, M.C. (2015) Assessing the effectiveness of the Birdsbesafe® anti-predation collar cover in reducing predation on wildlife by pet cats in Western Australia. *Applied Animal Behaviour Science,* **173**40-51.

Hernandez, S.M., Loyd, K.A.T., Newton, A.N., Carswell, B.L. & Abernathy, K.J. (2018) The use of point-of-view cameras (Kittycams) to quantify predation by colony cats (*Felis catus*) on wildlife. *Wildlife Research,* **45**(4)**,** 357-365.

Hervias, S., Oppel, S., Medina, F.M., Pipa, T., Diez, A., Ramos, J.A., de Ybanez, R.R. & Nogales, M. (2014) Assessing the impact of introduced cats on island biodiversity by combining dietary and movement analysis. *Journal of Zoology,* **292**(1)**,** 39-47.

Hodgens, P., Groffen, H., O’Handley, R., Vyas, A. & Lignereux, L. (2022) Cat predation of Kangaroo Island dunnarts in aftermath of bushfire. *Scientific Reports,* **12**(1)**,** 7272.

Hortelano-Moncada, Y.R.-R., A; Gil-Alarcón, G; Landeta-Solis, LJ; Vilchis-Conde, JM; Flores-Martínez, JJ; Rodríguez-Medina, R; Cervantes, FA (2024) Diet of free-ranging cats (*Felis silvestris catus*) and dogs (*Canis lupus familiaris*) in an urban ecological reserve in Mexico City. *Revista Mexicana de Biodiversidad,* **95**(2024).

Howes, C.A. (2002) Red in tooth and claw: 2 studies on the natural history of the domestic cat *Felis catus* Lin. in Yorkshire. *The Naturalist,* **127**101-130.

Hubbs, E.L. (1951) Food habits of feral house cats in the Sacramento Valley. *California Fish and Game,* **37**177-189.

Jensen, M.B., Willson, S.K. & Powell, A.N. (2022) How Effective Is the Birdsbesafe® Cat Collar at Reducing Bird Mortality by Domestic Cats? *Journal of Fish and Wildlife Management,* **13**(1)**,** 182-191.

Jones, E. (1977) Ecology of the Feral Cat, *Felis Catus* (L.), (Carnivora:Felidae) on Macquarie Island. *Wildlife Research,* **4**(3)**,** 249-262.

Karl, B.J. & Best, H.A. (1982) Feral cats on Stewart Island; their foods, and their effects on kakapo. *New Zealand Journal of Zoology,* **9**(2)**,** 287-293.

Kauhala, K., Talvitie, K. & Vuorisalo, T. (2015) Free-ranging house cats in urban and rural areas in the north: useful rodent killers or harmful bird predators? *Folia Zoologica,* **64**(1)**,** 45-55.

Kays, R.W. & DeWan, A.A. (2004) Ecological impact of inside/outside house cats around a suburban nature preserve. *Animal Conservation,* **7**273-283.

Kitts-Morgan, S.E., Caires, K.C., Bohannon, L.A., Parsons, E.I. & Hilburn, K.A. (2015) Free-ranging farm cats: home range size and predation on a livestock unit In northwest Georgia. *Plos One,* **10**(4)**,** e0120513.

Krauze-Gryz, D., Gryz, J. & Goszczynski, J. (2012) Predation by domestic cats in rural areas of central Poland: an assessment based on two methods. *Journal of Zoology,* **288**(4)**,** 260-266.

Krauze-Gryz, D., Zmihorski, M. & Gryz, J. (2017) Annual variation in prey composition of domestic cats in rural and urban environment. *Urban Ecosystems,* **20**(4)**,** 945-952.

Kutt, A.S. (2011) The diet of the feral cat (*Felis catus*) in north-eastern Australia. *Acta Theriologica,* **56**(2)**,** 157-169.

Lanszki, J., Kletečki, E., Trócsányi, B., Mužinić, J., Széles, G. & Purger, J. (2015) Feeding habits of house and feral cats (*Felis catus*) on small Adriatic islands (Croatia). *North-Western Journal of Zoology,* **12**.

Lepczyk, C. & Calver, M. (2022) Cat got your tongue? The misnomer of ‘community cats’ and its relevance to conservation. *Biological Invasions,* **24**.

Lockwood, H. (2024) The impact of predation by domestic cats (*Felis catus*) on British wildlife populations. PhD thesis, College of Science and Engineering, University of Derby.

Lockwood, H.L., Bulling, M. & Huck, M. (2025) What the Cat Dragged in: Quantifying Prey Return Rates of Pet Cats (Felis catus) With Outdoor Access in the UK. *Ecology and Evolution,* **15**(3)**,** e71063.

Loyd, K.A.T., Hernandez, S.M., Carroll, J.P., Abernathy, K.J. & Marshall, G.J. (2013) Quantifying free-roaming domestic cat predation using animal-borne video cameras. *Biological Conservation,* **160**183-189.

Matias, R. & Catry, P. (2008) The diet of feral cats at New Island, Falkland Islands, and impact on breeding seabirds. *Polar Biology,* **31**(5)**,** 609-616.

McDonald, J.L., Maclean, M., Evans, M.R. & Hodgson, D.J. (2015) Reconciling actual and perceived rates of predation by domestic cats. *Ecology and Evolution,* **5**(14)**,** 2745-2753.

McGregor, H., Legge, S., Jones, M.E. & Johnson, C.N. (2015) Feral cats are better killers in open habitats, revealed by animal-borne video. *Plos One,* **10**(8)**,** e0133915.

McMurry, F.B. & Sperry, C.C. (1941) Food of feral house cats in Oklahoma, a progress report. *Journal of Mammalogy,* **22**(2)**,** 185-190.

Medina, F.M., Garcia, R. & Nogales, M. (2006) Feeding ecology of feral cats on a heterogeneous subtropical oceanic island (La Palma, Canarian Archipelago). *Acta Theriologica,* **51**(1)**,** 75-83.

Medina, F.M., Lopez-Darias, M., Nogales, M. & Garcia, R. (2008) Food habits of feral cats (*Felis silvestris catus* L.) in insular semiarid environments (Fuerteventura, Canary Islands). *Wildlife Research,* **35**(2)**,** 162-169.

Medina, F.M., Melo, T., Oliveira, P., Nogales, M. & Geraldes, P. (2021) Trophic ecology of an introduced top predator (Felis catus) on a small African oceanic islet (Santa Luzia, Cabo Verde Islands). *African Journal of Ecology,* **59**(1)**,** 88-98.

Mella-Méndez, I., Flores-Peredo, R., Amaya-Espinel, J.D., Bolívar-Cimé, B., Mac Swiney G, M.C. & Martínez, A.J. (2022) Predation of wildlife by domestic cats in a Neotropical city: a multi-factor issue. *Biological Invasions,* **24**(5)**,** 1539-1551.

Millán, J. (2010) Feeding habits of feral cats *Felis silvestris catus* in the countryside of Majorca Island, Spain. *Wildlife Biology in Practice,* **6**(1)**,** 32-38.

Mitchell, J.C. & Beck, R.A. (1992) Free-ranging domestic cat predation on native vertebrates in rural and urban Virginia. *Virginia Journal of Science,* **43**197-208.

Morgan, S.A., Hansen, C.M., Ross, J.G., Hickling, G.J., Ogilvie, S.C. & Paterson, A.M. (2009) Urban cat (*Felis catus*) movement and predation activity associated with a wetland reserve in New Zealand. *Wildlife Research,* **36**(7)**,** 574-580.

Mori, E., Menchetti, M., Camporesi, A., Cavigioli, L., Tabarelli de Fatis, K. & Girardello, M. (2019) License to kill? Domestic cats affect a wide range of native fauna in a highly biodiverse Mediterranean country. *Frontiers in Ecology and Evolution,* **7**(477)**,** 1-11.

Nelson, S.H., Evans, A.D. & Bradbury, R.B. (2005) The efficacy of collar-mounted devices in reducing the rate of predation of wildlife by domestic cats. *Applied Animal Behaviour Science,* **94**(3-4)**,** 273-285.

Nilsson, N.N. (1940) The role of the domestic cat in relation to game birds in the Willamette Valley, Oregon. Oregon State College.

Nogales, M., Martin, A., Delgado, G. & Emmerson, K. (1988) Food spectrum of the feral cat (*Felis catus* L., 1758) in the juniper woodland on El Hierro (Canary Islands). *Bonner Zoologische Beiträge,* **39**(1)**,** 1-6.

Panchana, K., Herrera, I., Vargas, A., Mella-Méndez, I. & Flores-Peredo, R. (2025) Whiskers in the city: domestic cat predation in Ecuadorian coastal cities and associated factors. *Urban Ecosystems,* **28**(1)**,** 1-18.

Parmalee, P.W. (1953) Food habits of the feral house cat in East-Central Texas. *The Journal of Wildlife Management,* **17**(3)**,** 375-376.

Peck, D.R., Faulquier, L., Pinet, P., Jaquemet, S. & Le Corre, M. (2008) Feral cat diet and impact on sooty terns at Juan de Nova Island, Mozambique Channel. *Animal Conservation,* **11**(1)**,** 65-74.

Phillips, R.B., Winchell, C.S. & Schmidt, R.H. (2007) Dietary Overlap of an Alien and Native Carnivore on San Clemente Island, California. *Journal of Mammalogy,* **88**(1)**,** 173-180.

Piontek, A.M., Wojtylak-Jurkiewicz, E., Schmidt, K., Gajda, A., Lesiak, M. & Wierzbowska, I.A. (2021) Analysis of cat diet across an urbanisation gradient. *Urban Ecosystems,* **24**(1)**,** 59-69.

Read, J.L., Dagg, E. & Moseby, K.E. (2018) Prey selectivity by feral cats at central Australian rock-wallaby colonies. *Australian Mammalogy,* **41**(1)**,** 132-141.

Read, J.L., Moseby, K.E. & McGregor, H.W. (2024) Better to bluff than run: conservation implications of feral-cat prey selectivity. *Wildlife Research,* **51**(6)**,** -.

Ruxton, G.D., Thomas, S. & Wright, J.W. (2002) Bells reduce predation of wildlife by domestic cats (*Felis catus*). *Journal of Zoology,* **256**81-83.

Seymour, C.L., Simmons, R.E., Morling, F., George, S.T., Peters, K. & O’Riain, M.J. (2020) Caught on camera: the impacts of urban domestic cats on wild prey in an African city and neighbouring protected areas. *Global Ecology and Conservation,* **23**(2020)**,** e01198.

Shionosaki, K., Yamada, F., Ishikawa, T. & Shibata, S. (2015) Feral cat diet and predation on endangered endemic mammals on a biodiversity hot spot (Amami-Ohshima Island, Japan). *Wildlife Research,* **42**(4)**,** 343-352.

Smucker, T., Lindsey, G. & Mosher, S. (2000) Home range and diet of feral cats in Hawaii forests. *Pacific Conservation Biology,* **6**229-237.

Thomas, R.L., Fellowes, M.D.E. & Baker, P.J. (2012) Spatio-temporal variation in predation by urban domestic cats (*Felis catus*) and the acceptability of possible management actions in the UK. *Plos One,* **7**(11)**,** e49369.

Tschanz, B., Hegglin, D., Gloor, S. & Bontadina, F. (2011) Hunters and non-hunters: skewed predation rate by domestic cats in a rural village. *European Journal of Wildlife Research,* **57**(3)**,** 597-602.

van Heezik, Y., Smyth, A., Adams, A. & Gordon, J. (2010) Do domestic cats impose an unsustainable harvest on urban bird populations? *Biological Conservation,* **143**(1)**,** 121-130.

Weber, J.M. & Dailly, L. (1998) Food habits and ranging behaviour of a group of farm cats (*Felis catus*) in a Swiss mountainous area. *Journal of Zoology,* **245**(2)**,** 234-237.

Willson, S.K., Okunlola, I.A. & Novak, J.A. (2015) Birds be safe: Can a novel cat collar reduce avian mortality by domestic cats (*Felis catus*)? *Global Ecology and Conservation,* **3**359-366.

Woinarski, J.C.Z., Murphy, B.P., Legge, S.M., Garnett, S.T., Lawes, M.J., Comer, S., Dickman, C.R., Doherty, T.S., Edwards, G., Nankivell, A., Paton, D., Palmer, R. & Woolley, L.A. (2017) How many birds are killed by cats in Australia? *Biological Conservation,* **214**76-87.

Wood, V., Seddon, P.J., Beaven, B. & van Heezik, Y. (2016) Movement and diet of domestic cats on Stewart Island/Rakiura, New Zealand. *New Zealand Journal of Ecology,* **40**(1)**,** 186-190.

Woods, M., McDonald, R.A. & Harris, S. (2003) Predation of wildlife by domestic cats *Felis catus* in Great Britain. *Mammal Review,* **33**(2)**,** 174-188.

Yip, S.J.S., Dickman, C.R., Denny, E.A. & Cronin, G.M. (2014) Diet of the feral cat, *Felis catus*, in central Australian grassland habitats: do cat attributes influence what they eat? *Acta Theriologica,* **59**(2)**,** 263-270.

Yip, S.J.S., Rich, M.A. & Dickman, C.R. (2015) Diet of the feral cat, *Felis catus*, in central Australian grassland habitats during population cycles of its principal prey. *Mammal Research,* **60**(1)**,** 39-50.
